# Identification of Genomic Regions Associated with Concentrations of Milk Fat, Protein, Urea and Efficiency of Crude Protein Utilization in Grazing Dairy Cows

**DOI:** 10.3390/genes12030456

**Published:** 2021-03-23

**Authors:** Hewa Bahithige Pavithra Chathurangi Ariyarathne, Martin Correa-Luna, Hugh Thomas Blair, Dorian John Garrick, Nicolas Lopez-Villalobos

**Affiliations:** 1 School of Agriculture and Environment, Massey University, Palmerston North 4442, New Zealand; H.Blair@massey.ac.nz (H.T.B.); D.Garrick@massey.ac.nz (D.J.G.); N.Lopez-Villalobos@massey.ac.nz (N.L.-V.); 2 INRA, UE Herbipôle, F-63122 Saint-Genès-Champanelle, France; martin.correa.luna@gmail.com

**Keywords:** genome-wide association studies, candidate gene, single nucleotide polymorphism, efficiency of crude protein utilization, milk urea concentration, dairy cows

## Abstract

The objective of this study was to identify genomic regions associated with milk fat percentage (FP), crude protein percentage (CPP), urea concentration (MU) and efficiency of crude protein utilization (ECPU: ratio between crude protein yield in milk and dietary crude protein intake) using grazing, mixed-breed, dairy cows in New Zealand. Phenotypes from 634 Holstein Friesian, Jersey or crossbred cows were obtained from two herds at Massey University. A subset of 490 of these cows was genotyped using Bovine Illumina 50K SNP-chips. Two genome-wise association approaches were used, a single-locus model fitted to data from 490 cows and a single-step Bayes C model fitted to data from all 634 cows. The single-locus analysis was performed with the Efficient Mixed-Model Association eXpedited model as implemented in the SVS package. Single nucleotide polymorphisms (SNPs) with genome-wide association *p*-values ≤ 1.11 × 10^−6^ were considered as putative quantitative trait loci (QTL). The Bayes C analysis was performed with the JWAS package and 1-Mb genomic windows containing SNPs that explained > 0.37% of the genetic variance were considered as putative QTL. Candidate genes within 100 kb from the identified SNPs in single-locus GWAS or the 1-Mb windows were identified using gene ontology, as implemented in the Ensembl Genome Browser. The genes detected in association with FP (*MGST1*, *DGAT1*, *CEBPD*, *SLC52A2*, *GPAT4*, and *ACOX3*) and CPP (*DGAT1*, *CSN1S1*, *GOSR2*, *HERC6*, and *IGF1R*) were identified as candidates. Gene ontology revealed six novel candidate genes (*GMDS*, *E2F7*, *SIAH1*, *SLC24A4*, *LGMN*, and *ASS1*) significantly associated with MU whose functions were in protein catabolism, urea cycle, ion transportation and N excretion. One novel candidate gene was identified in association with ECPU (MAP3K1) that is involved in post-transcriptional modification of proteins. The findings should be validated using a larger population of New Zealand grazing dairy cows.

## 1. Introduction

Milk fat and protein play a vital role in New Zealand’s milk payment scheme [1], however, no attention is currently given to environmental traits such as nitrogen excretion. Protein utilization efficiency, measured as the proportion of dietary protein that is converted to protein in milk or protein in muscles, is influenced by gut microbes and is highly sensitive to the ratio of protein and fermentable energy in the diet [2]. If the diet is high in protein and relatively deficient in energy, such as most fresh pasture, surplus dietary protein results in the production of ammonia in the rumen and this is then converted to urea in the liver where it circulates in the blood supply. Urea in the blood is processed through the kidneys into the bladder then excreted in urine or diffused into the mammary gland as a component of milk. The concentration of urea in urine has a strong positive phenotypic correlation with concentration of MU [3] and is an indicator of protein utilization efficiency [2] in cows fed with total mixed rations. Efficiency of protein utilization can be defined in several ways [4]; (1) efficiency of crude protein utilization (ECPU): the ratio of crude milk protein yield (CPY) to dietary crude protein intake (CPI); (2) the crude protein balance (CPB): the difference between CPI and CPY, or (3) the residual protein intake (RPI): the difference between actual CPI and predicted CPI. The most widely used measure of protein efficiency is ECPU [4,5,6].

The heritabilities in New Zealand dairy cows for percentage of milk fat (from 0.62 to 0.66) and milk crude protein (0.67) [7] are moderate to high, MU (0.22) [8] is only moderate and ECPU (0.11) [9] is low. These estimates indicate that genetic changes for these traits are expected if these traits are placed under selection pressure. Changes in FP and CPP will directly influence farm profitability if they are not associated with differences in milk yield, whereas changes in MU and ECPU might at best indirectly affect farm profitability through their impact on animal efficiency or N emissions.

Genome-wide association studies (GWAS) scan the entire genome of an organism to discover associations between genetic markers and phenotypes. This is a crucial step in understanding the genes associated with the phenotype of interest. There are two major methods of GWAS depending on how the association between the marker and the trait is being tested. Single-locus GWAS is the simplest form of association testing and involves markers being fitted one at a time as fixed effects in a statistical model used to estimate the additive effect of the marker allele on the trait. The method relies on linkage disequilibrium (LD) between the markers being fitted and the causal mutation to find any evidence of association, unless the marker panel includes all sequence variants and other genomic features that represent the causal mutation. Testing the association of the phenotype with multiple markers fitted simultaneously as random effects is the other approach to GWAS. That approach takes advantage of the fact that linear functions of the multiple-markers being tested are in greater LD with the QTL in comparison to the single-locus GWAS approach and consequently, this increases the power of the experiment. Bayesian regression is one method of testing associations of multiple markers simultaneously with the phenotype.

Many studies have reported candidate genes associated with FP [10,11,12] and CPP [10,11,12,13] while a few have identified genomic regions associated with MU in dairy cattle fed total mixed rations in indoor circumstances [11,14,15]. Few GWAS have reported candidate genes associated with FP or CPP in New Zealand dairy cows [16,17,18]. There are no published GWAS in New Zealand that have identified candidate genes associated with MU nor are there any publications reporting GWAS on dairy cattle protein utilization efficiency, worldwide.

The objective of this study was to identify genes and QTLs associated with FP, CPP, MU or ECPU, using either a single-locus approach or a multi-locus single-step Bayes C approach.

## 2. Materials and Methods 

### 2.1. Animals and Phenotypes

Two herds comprising a total of 634 cows, from Massey University Dairy 1 and Dairy 4 farms, Palmerston North, New Zealand were used for this study. Test-day records from 467 cows milked in 2016–2017 and an overlapping group of 451 cows milked in the 2017–2018 production seasons were collected. These cows had a minimum of three herd-test records from lactations of not less than 150 days. Both herds comprised of mixed-breed Holstein Friesian (F) and Jersey (J) and F × J crossbred cows (Table 1).

Cows at Dairy 1 are managed in a once-a-day milking system with low levels of supplementary feeding and a low stocking rate (2.1 cows/ha). In contrast, cows in Dairy 4 are milked twice a day, with high levels of supplementary feeding and a higher stocking rate (2.8 cows/ha).

Herd-test records on FP and CPP for each cow were collected on a monthly basis. Daily crude protein yield (CPY) was estimated at each month as the product of daily CPP and corresponding daily milk yield. The MU concentration for each cow was predicted by mid-infrared spectral data from additional milk samples collected only for three of the herd-tests per season, representing early (September), mid (December) and late (March) lactation. A daily composite sample of morning and afternoon milking followed by weighting according to morning and afternoon milk yields were used for estimating daily MU when twice-a-day milking was practiced, whereas the single sample was used when once-a-day milking was practiced on the sampling days. Milk samples were measured for MU at MilkTestNZ (Hamilton, New Zealand) using the CombiFossTM 7 instrument (Foss Electric, Hillerød, Denmark).

Dietary metabolizable energy (DME) and dietary crude protein percentage (DCPP) were estimated by analyzing pasture, crop and supplementary feed offered to the cows using near-infrared spectroscopy. The total metabolizable energy (ME) requirements of each cow on each day of lactation was calculated as the sum of the estimated ME requirements for maintenance, pregnancy, lactation and live weight change, as described by Correa-Luna et al. [19]. Apparent daily dry matter intake was calculated as the estimated daily total ME requirement of each cow divided by total DME offered to the cow on the corresponding day. Daily crude protein intake (CPI) of the cow was determined as DCPP available in any diet offered to the cow multiplied by the estimated daily DMI. Daily ECPU was estimated as the proportion of CPY in relation to estimated CPI. Daily estimates of milk yield, FP and CPP were predicted by modeling the herd-test records of cows using third order orthogonal polynomials for each lactation [19]. The estimated correlation coefficients of actual and predicted values were greater than 0.98.

### 2.2. Genotypes and Quality Control

Phenotypes were collected from a total of 634 cows but only a subset of 490 cows were genotyped because sample collection for genotyping was done after the 2017–2018 production season and 114 of the cows milking in 2016–2017 had already been culled. DNA was extracted from the ear punch tissue samples which were then genotyped using Bovine Illumina 50K SNP-chips. SNP & Variation Suite (SVS 8.8 [20]) was used for initial analysis and quality control (QC) steps. The genotypes recorded in Illumina A/B allele format were converted to 0, 1 or 2, depending on the number of B alleles present at each locus. Loci with a call rate ≤ 80% or minor allele frequency ≤ 0.01, as well as animals with a call rate ≤ 80% were excluded from the data set. After these QC steps, a total of 45,062 SNPs qualified for association analysis.

### 2.3. Descriptive Statistics

Descriptive statistics for milk composition traits (FP, CPP, MU) and ECPU were performed for 634 cows using the MEANS procedure of SAS package 9.4 (SAS Institute Inc. 2013, Cary, NC, USA).

### 2.4. Parameter Estimation

All the 634 cows with phenotypes were used for estimating variance components. Variance components for animal additive genetic variance (σ^2^_a_), within lactation permanent environment variance (σ^2^_pw_), across lactation permanent environment variance (σ^2^_pa_) and residual effects variance (σ^2^_e_) were estimated by fitting the following repeatability animal model as implemented in the JWAS package 0.7.3 [21]:

**y = Xb + Zu + Wp + Kc + e**, (1)

where **y** is the vector of test-day phenotypes, **X** is the design matrix of fixed effects, **b** is the vector of fixed effects which included contemporary group defined by herd-test-date (HTD), parity, days in milk nested within each HTD, regression on proportion of **F**, and regression on F × J heterosis, **Z** is the design matrix of random animal additive genetic effects (**u**), **W** is the design matrix of within lactation permanent environment effects (**p**), **K** is the design matrix of across lactation permanent environment (**c**) effects and **e** is the vector of random residual errors. It was assumed that the animal additive genetic effects were distributed as **u**~N(0, **A**σ^2^_a_) where **A** is the numerator relationship matrix, **p**~N(0, **I**_1_σ^2^_pw_) where **I**_1_ is an identity matrix of order equals to the number of interactions between cows and production seasons; **c**~N(0, **I**_2_σ^2^_pa_) where **I**_2_ is an identity matrix of order equals to the number of cows with records, and **e**~N(0, **I**_3_σ^2^_e_) where **I**_3_ is an identity matrix of order equal to the total number of test-day records.

The trait heritabilities (h^2^) and repeatabilities (t) were calculated using the estimated variance components. The heritability for a trait was calculated as the proportion between the genetic variance and the phenotypic variance. The phenotypic variance was calculated as the sum of σ^2^_a_, σ^2^_pw_, σ^2^_pa_ and σ^2^_e_. The repeatability of the trait was calculated as the proportion of permanent variance (the sum of σ^2^_a_, σ^2^_pw_, and σ^2^_pa_) and the phenotypic variance.

A single-step Bayes C linear mixed model (genomic data), as implemented in the JWAS package 0.7.3, was used to account for variance explained by SNP markers. The model used for estimating individual SNP effects was the same as model (1), other than the inclusion of random SNP effects that were fitted simultaneously using Bayes C priors (model 2). There is a model equation corresponding to genotyped animals and another one for non-genotyped animals. The following model equation was fitted for genotyped animals and included marker effects:

**y = Xb + Zu + Wp + Kc + Ms + e**, (2)

where **M** is the design matrix of genotypes corresponding to the number of genotyped loci (each coded as 0, 1, 2), and **s** is a vector of marker effects. It was assumed that the animal additive genetic effects are distributed as **u**~N(0, **A**σ^2^_g_) where **A** is the numerator relationship matrix for polygenic effects and σ^2^_g_ is the additive genetic variance not explained by markers, representing some fraction (1–d) of σ^2^_a_ from the pedigree-based best linear unbiased prediction model. The priors for marker effects have identical and independent mixture distributions, where each has a point mass at zero with probability π (marker effect is null), or a univariate-normal distribution with a null mean and variance σ^2^_s_, with probability 1 − π such that the scalar variance of the vector of genomic breeding values, i.e., var(**Ms**) reflects the additive-genetic variance explained by markers (i.e., dσ^2^_a_). The mixed model equations for the non-genotyped individuals was similar to model (2), except, **M** contains the imputed genotypes of non-genotyped individuals in this analysis instead of actual genotypes for genotyped individuals. Since this model deals with imputed genotypes, the model equation includes another matrix, **H** and vector **m,** where **H** is the incidence matrix corresponding to the imputation residuals and **m** is a vector of imputation residual effects.

Pre-estimated values for σ^2^_a_, σ^2^_pw_, σ^2^_pa_ and σ^2^_e_ were those obtained fitting the repeatability model of Equation (1) and were considered as prior variances in estimating marker effects. The SNPs with non-zero effects in Bayes C have a common variance [22] denoted σ^2^_s_ whose prior was estimated as described by Fernando and Garrick [23] (model 3):

(3) $$\sigma_{s\;}^{2}=\frac{{d\sigma }_{a}^{2}}{\;k\;\left(1\;-\;\pi \right)\;2\mathrm{pq}\;},$$

where $\sigma_{a}^{2}$ is the additive genetic variance of the trait, d is the proportion of additive genetic variance assumed explained by markers, k is the number of markers from the SNP-chip that passed QC, π is the probability that the markers have null effects, 2pq is the average heterozygosity across all genotyped loci.

The assumed proportion of SNPs with zero effect (π) was 0.997 for all the traits [24], corresponding to about 135 SNPs (1 − 0.997 = 0.003 of 45,062 SNPs) fitted per iteration. The value for π was selected to be 0.997 to fit fewer SNPs in the model than number of animals and this results in less shrinkage of marker effects towards zero than would occur when more markers are fitted [23]. The SNP genotypes with sampled non-zero effects on the traits were fitted in the model along with phenotypes and pedigrees in a single-step in any particular sample that comprises the Markov chain. A three-generation pedigree containing 180 sires, 500 dams was included in the models to infer covariances between animals for the additional polygenic effect and imputation residual effects.

Markov chain Monte Carlo (MCMC) comprised 50,000 iterations and marker effects from each 50th iteration were used to estimate the genomic merit of all animals. The samples for genomic merit were obtained in each MCMC iteration by multiplying the matrix **M** by the corresponding sample of marker effects **s** for that MCMC iteration. The posterior mean of variances (σ^2^_m_) of the sampled genomic breeding value for each iteration across all cows was used to estimate the genomic heritability (s^2^) which is the proportion of phenotypic variance explained by the additive effects of the SNPs (i.e., σ^2^_m_ is dσ^2^_a_). After including SNP effects in the model, heritabilities and repeatabilities of the traits were re-estimated by replacing σ^2^_a_ in the numerator and the denominator of the ratio with σ^2^_g_ + σ^2^_m_.

### 2.5. GWAS

#### 2.5.1. Single-Locus Method

Cows (n = 490) with both phenotypes and genotypes were used for single-locus genome-wide association studies and the analyses were performed in two steps. In step 1 each test-day record for each trait was adjusted for non-genetic fixed effects, within and across lactation permanent environment of the cow to account for repeated measures on the same animal using the regression coefficients of **b**, **p** and **c** estimated in model (1), respectively, in ASReml package 4.1 [25]. The phenotype file for association analysis contained multiple rows for each adjusted test-day phenotype of the cow including a different number of rows corresponding to each test-day record. In step 2, association of the adjusted phenotypes in step 1 (e*) with the SNP genotypes was calculated in the SVS software (8.8), using the following additive, single-locus, mixed-model called Efficient Mixed-Model Association eXpedited (EMMAX) (with the variable definitions as in model 2):

e* = Zu + Ms + e, (4)

In this model, the association was tested for each SNP fitted separately as a fixed effect. A genomic relationship matrix (**G**) was computed in the form of identity-by-state using the same genotypes analyzed which was scaled using Gower’s centering approach as described by Kang et al. [26] and fitted as a random effect in the model to account for structure due to relatedness among animals. This was done by defining a scaling factor (W) as follows:

(5) $$w\;=\frac{n-1}{\;\mathrm{Trace}\left(\mathrm{CGC}\right)\;},$$

and multiplying by **G** to obtain scaled **G** (**G**_s_) as **G**_s_ = **WG**, where n is the number of cows, **C** = **I** − 11′/n and 1 is a length n vector of ones, and **I** is the identity matrix related to the number of cows.

The estimated associations were presented as Manhattan plots in which −log_10_(*p*-values) were plotted against the genomic locations of the markers using the annotation for bovine assembly UMD3.1 in SVS (8.8) software. The estimated marker associations were corrected for multiple testing using Bonferroni corrections [27]. The genome-wide threshold was estimated using a nominal *p*-value of 0.05 (0.05/number of SNPs), and the suggestive threshold was estimated using a nominal *p*-value of one (1/number of SNPs). The Bonferroni corrected *p*-value threshold for genome-wide significant level was 1.11 × 10^−6^ (0.05/45,062) which corresponded to 5.95 on a −log_10_(*p*-value) scale. The *p*-value threshold for suggestive associations was 2.22 × 10^−5^ (1/45,062) which corresponded to a −log_10_(*p*-value) of 4.65. The SNPs that surpass the Bonferroni adjusted genome-wide *p*-value were described and their genomic position, allele substitution effect and closest gene were reported.

#### 2.5.2. Single-Step Bayesian Method

Genome-wide association analyses were performed separately for each milk composition trait and each of ECPU trait using a Bayes C linear mixed model as implemented in the JWAS package 0.7.3 [21] including both genotyped (n = 490) and non-genotyped (n = 144) cows in a single step model (model 2). The MCMC included 50,000 iterations. The statistical models used were similar to model 2 (including the same fixed and random effects). Variances (σ^2^_a_, σ^2^_pw_, σ^2^_pa_, σ^2^_e_ and σ^2^_m_) obtained from the model Equation (1) were taken to be the prior variances in this analysis. The probability that SNPs would have zero effect on the trait was chosen to be 99.7% (π = 0.997). The SNP genotypes with non-zero effects in any particular iteration were simultaneously fitted in the model in that iteration along with phenotypes and pedigree in a single step.

Marker effects were collectively used to predict the genomic merit of cows in chromosomal regions that include non-overlapping 1 Mb windows based on the physical map order [28,29]. Gibbs samples of marker effects within each 1 Mb window were used every 50th iteration to sample the genomic merit of all cows for every window. The window genomic merit was sampled by multiplying the genotype matrix related to number of minor alleles for each marker within each 1 Mb window by their corresponding marker effects of each MCMC iteration. The variance associated with genomic merit of each 1-Mb window across all cows was expressed as a proportion of variance associated with whole-genomic prediction samples to identify the most informative genomic regions. There were 2676 unique SNP windows across the whole genome including 31 chromosomes (29 autosomes, mitochondrial, X chromosome). The expected proportion of variance explained (PVE) by each 1 Mb window was 3.74 × 10^−2^% (1/2676 SNPs) and window explained at least 0.19% of genetic variance, which corresponds to 5 times the expected proportion of variance (3.74 × 10^−2^% × 5 = 0.19%), was considered as a suggestive significance level [30] while the SNP windows that explained 0.37% of genetic variance, which corresponds to 10 times the expected proportion of variance (3.74 × 10^−2^% × 10 = 0.37%), were considered to reach genome-wide significance level. The SNP windows that surpass the genome-wide significance level were considered as putative QTL (used for further analysis) and their genomic position, number of SNPs in each window, PVE (the genetic variance explained by each window divided by the genetic variance explained by the whole genome), window posterior probability of association (WPPA; the posterior probability that marker regression coefficient is non-zero for at least one SNP in a window) and associated genes were reported. The estimated proportion of genetic variance explained by each 1 Mb window was plotted against genomic locations of the markers using the qqman package in R software 3.6.2 [31].

### 2.6. Candidate Genes and Functional Analysis

Each individual SNP that either surpassed the genome-wide or suggestive significance level was examined to locate the closest genes within 100 kb upstream and downstream from the identified SNP in the single-locus method. Each individual 1-Mb genomic window that either surpassed the genome-wide or suggestive significance level was examined to identify the closest genes including those within 100 kb upstream or downstream from the identified window. All genes were identified using the *Bos taurus* reference genome in Ensembl (Ensembl release 94), using the UMD 3.1 assembly (https://www.animalgenome.org/cgi-bin/QTLdb/index/, accessed on 10 June 2020). The biological functions of significant genes were identified using the gene ontology (GO tool [32]) as implemented in Ensembl (Ensembl release 94).

## 3. Results

### 3.1. Descriptive Statistics

Descriptive statistics for the test-day milk composition and ECPU are presented in Table 2.

### 3.2. Parameter Estimation

Estimates of variance components, heritabilities and repeatabilities of milk percentage traits, milk urea and efficiency of crude protein utilization using a univariate repeatability animal model are presented in Table 3.

The estimated variance components, heritabilities and repeatabilities of the traits applying the univariate single-step Bayesian (Bayes C, π = 0.997) linear mixed model are presented in Table 4.

The s^2^ of traits varied from zero to moderately high; s^2^ of ECPU was near zero, s^2^ of MU was low and s^2^ of milk percentage traits were moderate.

### 3.3. GWAS

#### 3.3.1. Single-Locus Method

**Figure 1:**
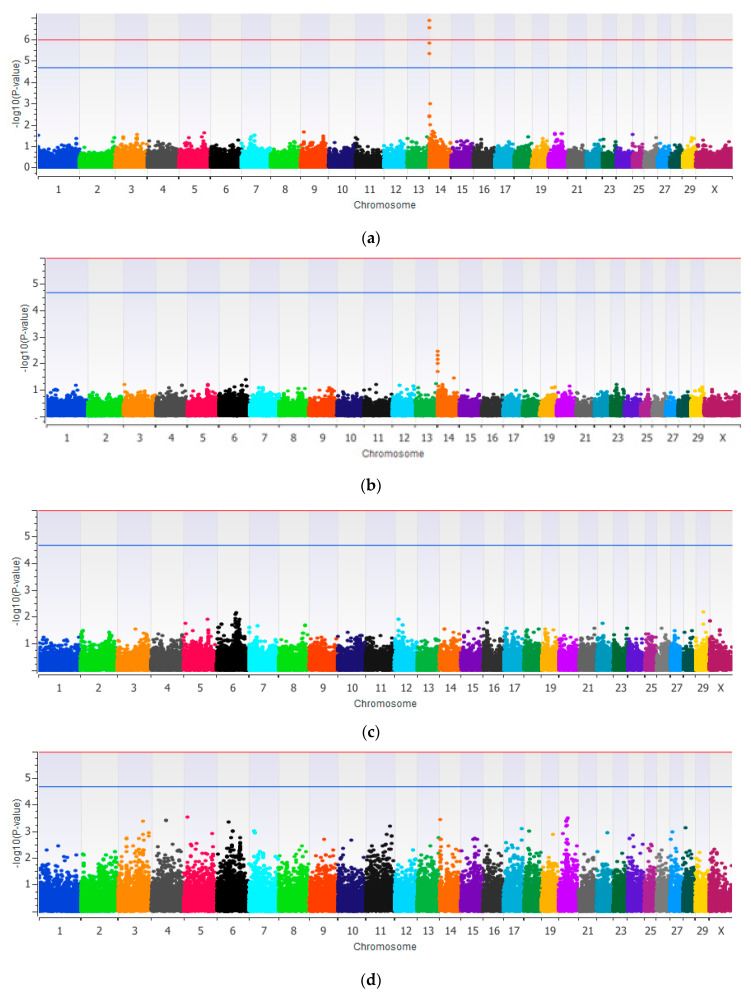
Manhattan plot showing genome-wide associations for fat percentage (**a**), crude protein percentage (**b**), milk urea concentration (**c**) and efficiency of crude protein utilization (**d**). The red line shows the genome-wide significant level at −log_10_(*p*-value) = 5.95 and the blue line shows the suggestive association significant level at −log_10_(*p*-value) = 4.65.

Manhattan plots resulting from the single-locus EMMAX method are presented in Figure 1 and details of the SNPs that surpassed the genome-wide significance threshold *p*-value for FP are presented in Table 5. Two SNPs from chromosome 14 were found to be significantly associated (*p* < 1 × 10^−6^) with FP at genome-wide significant threshold (Figure 1a). No SNPs were significantly associated with CPP, MU, and ECPU either at genome-wide significance threshold or the suggestive threshold (Figure 1b–d, respectively). The SNPs that surpass the suggestive *p*-value were described and their genomic position, allele substitution effect, and genes were reported as Supplementary Materials (Table S1).

#### 3.3.2. Candidate Genes and Functional Analysis

Two candidate genes were identified to be associated with FP; diacylglycerol O-acyltransferase 1 (*DGAT1*) and solute carrier family 52 member 2 (*SLC52A2*) (Table 5). Two suggestive genes in association with FP were reported; maestro heat-like repeat family member 1 (*MROH1*), and cleavage and polyadenylation specific factor 1 (*CPSF1*) (Table S1).

#### 3.3.3. Single-Step Bayesian Method

**Figure 2:**
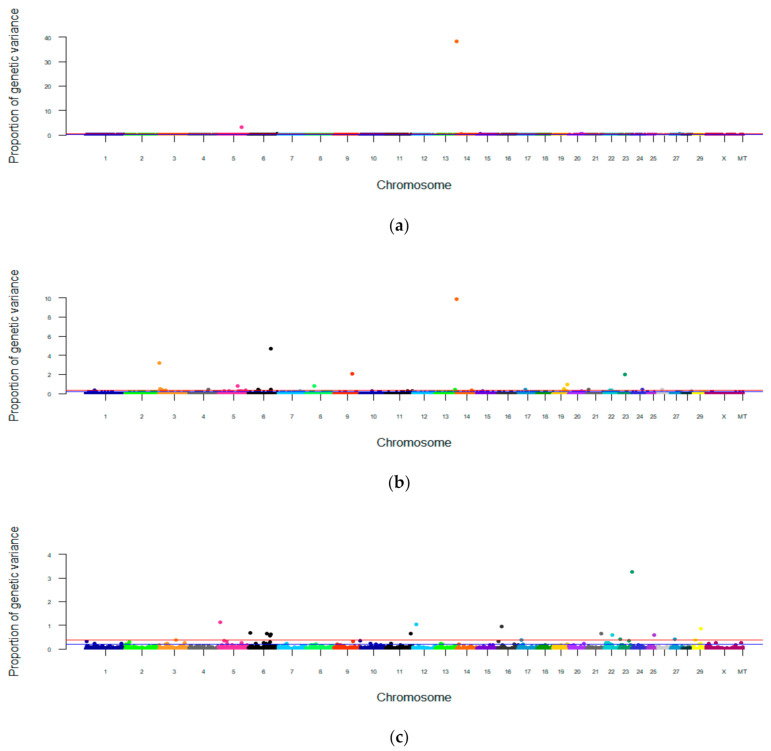

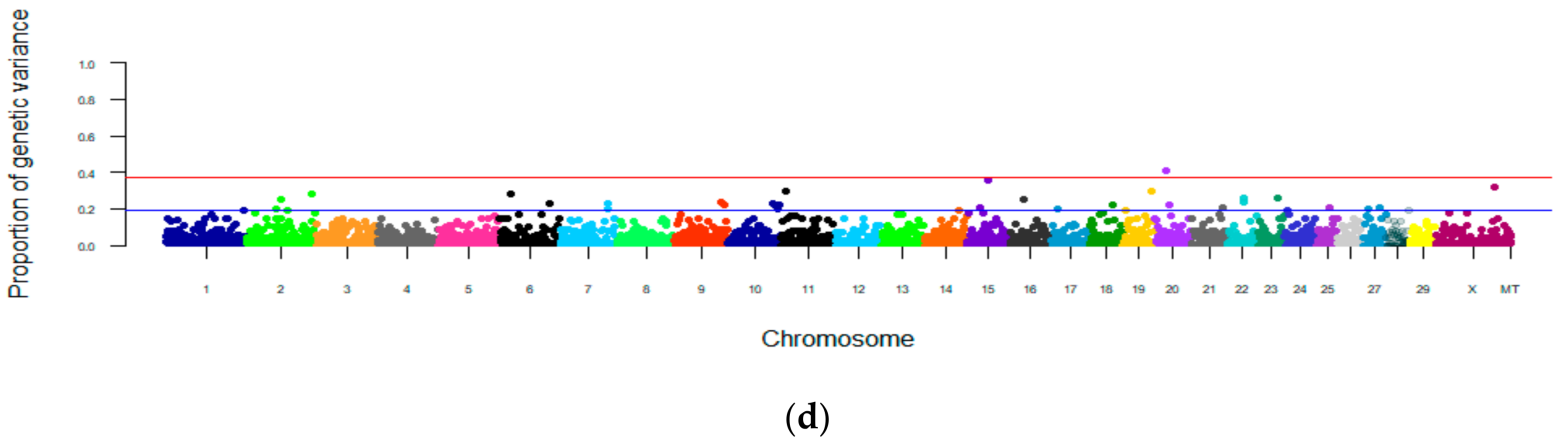
Manhattan plot showing genome-wide association window variance for fat percentage (**a**), crude protein percentage (**b**), milk urea concentration (**c**), and efficiency of crude protein utilization (**d**). The red line shows the genome-wide significant level at PVE = 0.37% and the blue line shows the suggestive association significant level at PVE = 0.19%.

The Manhattan plots based on a single-step Bayesian method are presented in Figure 2. Details of the 1 Mb SNP windows that surpassed the genome-wide significance threshold (PVE ≥ 0.37%) are presented in Table 6. The SNP windows that surpassed the suggestive significance level were presented as additional information in Supplementary Materials (Table S2). The number of SNPs included in a 1 Mb window varied from 1 to 99 and averaged 17 SNPs per window (±5.73).

Eight 1 Mb SNP windows from chromosomes 5, 6, 14, 15, 20 and 27 were significantly (PVE ≥ 0.37%) associated with FP (Figure 2a). The proportion of genetic variance explained by the most significant 1 Mb window was 38% and the windows that were significantly associated with FP at the genome-wide threshold collectively explained 44% of the total genetic variance (Table 6).

Twenty 1 Mb SNP windows from 14 different chromosomes, including 3, 4, 5, 6, 8, 9, 13, 14, 17, 19, 21, 23, 24 and 26, were significantly (PVE ≥ 0.37%) associated with CPP (Figure 2b). The proportion of genetic variance explained by the QTLs varied from 0.38 to 9.83% and all windows that were significantly associated with CPP at genome-wide threshold collectively explained approximately 39% of the total genetic variance (Table 6).

Eighteen 1 Mb SNP windows from 14 different chromosomes, including 3, 5, 6, 11, 12, 16, 17, 21, 22, 23, 25, 27 and 29, were significantly (PVE ≥ 0.37%) associated with MU (Figure 2c). The top QTL spans in chromosome 23 (WPPA = 0.7) alone explained 3.25% of total genetic variance of MU. The QTLs that were significantly associated with MU at the genome-wide threshold collectively explained only 14% of the total genetic variance (Table 6).

Only one 1 Mb SNP window from chromosome 20 was significantly (PVE ≥ 0.37%) associated with ECPU (Figure 2d). The PVE explained by significant QTL was only 0.41% (Table 6).

#### 3.3.4. Candidate Genes and Functional Analysis

The significant genomic windows harbored good candidate genes for FP including; *DGAT1*, glycerol-3-phosphate acyltransferase 4 (*GPAT4*), microsomal glutathione S-transferase 1 (*MGST1*), acyl-CoA oxidase 3, pristanoyl (*ACOX3*), and CCAAT enhancer binding protein delta (*CEBPD*). These genes were located at chromosome 6, 14 and 27 (Table 6). A suggestive gene reported to be associated with FP was glutamic-pyruvic transaminase 2 (*GPT2*) (Table S2).

Most of the significant genomic windows contained good candidate genes for CPP, including; *DGAT1*, α-S1-casein (*CSN1S1*), β-casein (*CSN2*), α-S2-casein (*CSN1S2*), kappa-casein (*CSN3*), Bos taurus ribosomal protein S12 (*RPS12*), glutamate ionotropic receptor NMDA type subunit 2C (*GRIN2C*), eukaryotic translation initiation factor 3 subunit D (*EIF3D*), ADAM metallopeptidase domain 11 (*ADAM11*), the golgi SNAP receptor complex member 2 (*GOSR2*), HECT and RLD domain containing E3 ubiquitin protein ligase family member 6 (*HERC6*), and insulin-like growth factor 1 receptor (*IGF1R*). These genes were located on chromosomes 5, 6, 9, 14, 19 and 21 (Table 6). Two genes associated with CPP at suggestive level were tripartite motif containing 45 (*TRIM45*) and ubiquitin protein ligase E3 component n-recognin 5 (*UBR5*) (Table S2).

Six good candidate genes for MU were found over 18 genomic windows and were located in chromosomes 5, 11, 12, 21 and 23 (Table 6). The candidate genes associated with MU were GDP-mannose 4,6-dehydratase (*GMDS*), E2F transcription factor 7 (*E2F7*), solute carrier family 52 member 2 (*SIAH3*), solute carrier family 24 member 4 (*SLC24A4*), legumain (*LGMN*) and argininosuccinate synthase 1 (*ASS1*). Twenty genes were identified to be associated with MU at suggestive significant level (Table S2).

The 1-Mb genomic window that was significantly associated with ECPU harbored one good candidate gene on chromosome 20, Mitogen-activated protein kinase 1 (*MAP3K1*) (Table 6). Sixteen genes were identified to be associated with ECPU at suggestive significant level (Table S2). These genes include, mitogen-activated protein kinase kinase kinase kinase 4 (*MAP4K4*), golgi-associated, γ adaptin ear containing, ARF binding protein 3 (*GGA3*), tripartite motif containing 63 (*TRIM63*) and CDP diacylglycerol synthase 1 (*CDS1*).

## 4. Discussion

### 4.1. Descriptive Statistics

The ranges of FP (from 1.77 to 9.77%) and CPP (from 2.72 to 7.66%) found in the current study were within the ranges (1.84 to 11.32% and 2.76 to 6.77%) previously reported by Sneddon et al. [33]. The higher CV of FP and CPP found in this study is likely attributed to having cows milked once and twice a day. Cows milked once a day are known to produce milk with higher fat and protein percentages than cows milked twice a day [34]. The average MU concentration (29.96 mg/dL) observed by Beatson et al. [8] in mixed-breed New Zealand dairy cows was somewhat higher than the average MU (25.6 mg/dL) observed in the current study. Beatson et al. [8] analyzed cows from 540 herds located throughout New Zealand for four lactations, therefore, their MU predictions should be a better representative of average MU in New Zealand dairy cows than our predictions based on 634 cows on just two farms at Massey University. Moreover, the twice-a-day-milking herd in the current study receives more supplementary feed than an average New Zealand herd [35], which would likely improve the protein utilization efficiency of those cows, resulting in lower average MU. The reported average MU in this study was comparable with the range (22.6 to 25.57 mg/dL) found in overseas studies [36,37]. The average ECPU (24.4%) observed in this study is consistent with the average protein utilization efficiency (27%) and nitrogen utilization efficiency (27.7%) reported by Zamani et al. [4] and Huhtanen et al. [6], respectively. Overall, the data obtained from these herds appear representative of cows in New Zealand.

### 4.2. Parameter Estimation

The estimates of h^2^ and t were consistent with the literature for FP and CPP [7] for MU [8,38] and for ECPU [4]. The s^2^ for milk percentage traits was moderate, indicating good marker predictions for FP and CPP traits; s^2^ for MU was low, indicating comparatively low marker prediction for MU; and s^2^ of ECPU was zero, indicating poor marker prediction for ECPU. The s^2^ reported for FP (0.31) in this study is comparable with the estimate (0.35) found in another New Zealand study using a much larger dataset comprising mixed-breed cows [17]. The greater s^2^ range reported for CPP (from 0.59 to 0.62) in Kemper et al. [39] in multi-breed cows of Australia using a nonlinear Bayesian method in comparison to the estimated s^2^ in this study (0.38), could be a benefit of using high-density arrays (777K SNPs) for genotyping of their cows. The estimated s^2^ in the current study for MU (0.13) is comparable to estimated s^2^ (0.14) by Bouwman et al. [14] in Dutch Holstein Friesian cows. The estimates for the s^2^ for all traits indicated that the markers explained considerable proportions of phenotypic variance of the traits, except for ECPU. The current study used 45K SNPs and therefore, the number of SNPs within causative gene is probably limited. However, the higher proportion of genetic variance explained by markers for milk constituents in the current study despite the small sample size, suggests there is considerable linkage disequilibrium among SNPs and causative genes.

### 4.3. GWAS

#### 4.3.1. Single-Locus Method

Two SNPs in chromosome 14 were significantly associated with the FP at the genome-wide significant threshold, suggesting that these two SNPs might be in linkage disequilibrium with the QTL for FP. None of the SNPs for CPP, MU, and ECPU reached significance at the genome-wide threshold. This observation suggests that these traits are under the control of many genes, each with small effects which could not be detected with either the single-locus method or with the small number of animals available in this study.

#### 4.3.2. Candidate Genes and Functional Analysis

The single-locus method (EMMAX) identified two candidate genes (*DGAT1* and *SLC52A2*) associated with FP. The association of *DGAT1* with FP is widely reported [40,41,42,43] and the gene has biological functions of diacylglycerol metabolic process (GO:0046339), fatty acid homeostasis (GO:0055089), lipid storage (GO:0019915), long-chain fatty-acyl-CoA metabolic process (GO:0035336) and triglyceride biosynthetic process (GO:0019432). The *DGAT1* gene is also associated with very-low-density lipoprotein particle assembly in the liver (GO:0034379). The aggregation of lipoprotein in the liver subsequently causes fatty liver in lactating dairy cows [44]. This process may avoid the mobilized fatty acids being used efficiently by the cow in an attempt to meet the demands of either maintenance by being oxidized or milk fat production [44]. The gene *SLC52A2* has an assigned function of transportation of riboflavin (GO:0032218). Riboflavin is involved in the cellular metabolism of fats and proteins; therefore, the activity of this gene may affect the utilization of feed fats and proteins and ultimately this balances the proportion of feed constituents that converted into milk constituents. The relationships of *SLC52A2* gene with FP of Chinese Holstein cows were reported recently by Wang et al. [42] (2020) in a GWA study.

Two suggestive genes associated with FP were reported. The gene *CPSF1* has the biological function of mRNA polyadenylation (GO:0006378) and its association with milk production in Canadian Holstein Friesians was reported in a study by Nayeri et al. [45]. The *MROH1* gene has no known biological function but their relationships with FP was reported [42].

#### 4.3.3. Single-Step Bayesian Method

Collectively, the relatively modest number of windows significantly associated with FP at the genome-wide threshold explain about 43% of the total genetic variance, this confirms the initial idea that a small number of genes have important roles in regulating FP. The top-most windows in this method and the top-most significant SNP in single-locus method were located within the DGAT1 gene. This indicates that both methods perform well when the effect of the gene is large. However, more genomic regions were identified by the single-step Bayesian method for FP than by the single-locus method. No SNPs reached the level of significance for CPP, MU and ECPU traits when the associations were tested using the single-locus method. However, some genomic windows for CPP, and MU, and one genomic window for ECPU, which harbored good candidate genes, became significant at the genome-wide level when the associations were tested using the Bayesian method. This was likely due to the greater power of detection of associations when the markers were fitted simultaneously as random effects in the model and therefore, more genetic variation was captured by the markers in comparison to single-locus methods [23]. The PVE by the significant window of ECPU was only 0.41%, suggesting again that the trait is polygenic with no genes of major effect. In a previous study [46], feed efficiency was identified as a polygenic trait in multi-breed cow herds in Australia, which had a moderately high h^2^ (0.36) and was under control of a larger number of SNPs than found here. Therefore, it is not surprising the observed very low genetic variance from the significant window for ECPU in the current study.

#### 4.3.4. Candidate Genes and Functional Analysis

Under the assumptions applied for the single-step Bayes C method in the current study, five, twelve, six and one gene associated with FP, CPP, MU and ECPU were identified, respectively. The *DGAT1*, *MGST1*, *GPAT4*, *ACOX3* and *CEBPD* genes were identified as being important for FP. The functions of *DGAT1* were described earlier. Another important gene associated with FP is *MGST1*, which is related to cellular response to lipid hydroperoxide (GO:0071449). This gene was identified as a causative gene for FP in New Zealand mixed-breed dairy cows, although the functional relationship of the gene with FP has not yet been revealed [17]. The gene *GPAT4* is involved in the fatty acid metabolic process (GO:0006631) and mammary gland development (GO:0030879). Genome-wide association studies have shown that this gene is highly polymorphic and highly significantly associated with FP in German Holstein Friesians [47,48]. The *ACOX3* gene is also involved with the fatty acid metabolic process (GO:0006631) and GWAS has shown its relationship with fatty acids synthesis in Canadian Holstein cows [49]. The gene, *CEBPD* is related with fat cell differentiation (GO:0045444), this process enhances synthesis and storage of fat. The suggestive gene *GPT2* has a biological function related to regulation of biosynthesis (GO:0009058), therefore, the gene might be important in milk and milk component production. However, its association with FP in dairy cows has not yet been revealed through GWAS. Most of the genes associated with FP in the current study have previously been reported as candidate genes for FP, this gives confidence that the current findings could be applied in the selection of genetically superior New Zealand dairy cows.

Twelve candidate genes; *DGAT1*, *CSN1S1*, *CSN1S1*, *CSN1S2*, *CSN3*, *RPS12*, *GRIN2C*, *EIF3D*, *ADAM11*, *GOSR2*, *HERC6* and *IGFR1* were identified as candidate genes for CPP. The casein cluster genes *CSN1S1*-*CSN2*-*CSN1S2*-*CSN3* encode αs1, β, αs2 and κ caseins, respectively. Casein is the most common type of protein in bovine milk and these genes are known to significantly affect the physical, chemical and the nutritional quality of milk [50]. The genes in the casein family are over-expressed in the mammary gland compared to other tissues [51]. Additionally, *CSN1S1* (GO:0050821) and *CSN3* (GO:0050821) genes are involved in the stabilization of encoded proteins by preventing their degradation. The association of these genes with milk protein composition has been reported in lactating dairy cows in many studies including Zhou et al. [52] and Sanchez et al. [53]. The *RPS12* gene encodes the ribosomal protein (GO:0006412), which is a component of the 40S subunit of the ribosome (GO:0022627). Ribosomes are organelles that synthesize protein within the cell. This gene plays a vital role by coding for a structural constituent of the ribosome, which makes it highly likely to be an important candidate gene for milk protein synthesis. Gene *GRIN2C* has a known biological function of negative regulation of protein catabolic process (GO:0042177). The gene *EIF3D* codes for 3 Subunit D, which initiates the protein translation through mTOR signaling pathway [54]. This pathway is known to positively control milk protein synthesis in ruminants [55]. The *ADAM11* gene is related to proteolysis (GO:0006508), the hydrolysis of proteins into smaller polypeptides and/or amino acids by cleavage of their peptide bonds. The *GOSR2* gene has a biological function related to protein transport (GO:0015031). This gene has been identified as a candidate gene for milk protein percentage in a meta-analysis in lactating cows [56]. The *HERC6* is a gene related to protein ubiquitination (GO:0016567). This gene has been suggested as candidate gene for protein yield [57] and lactation persistency in Canadian Holstein cattle [58]. The gene *IGF1R* is involved with protein phosphorylation (GO:0016310). This gene is associated with milk protein yield in four dairy breeds [59], Simmental cows in Poland [60]. Both suggestive genes; *TRIM45* and *UBR5* have biological function related to protein ubiquitination (GO:0016567). Some of the genes identified in association with CPP in this study were previously reported as good candidate genes using GWAS. This study identified additional genes associated with CPP in New Zealand dairy cows which can be potential candidate genes for the trait.

The genes found to be in association with MU, *GMDS*, *E2F7*, *SIAH1*, *SLC24A4*, *LGMN*, and *ASS1,* are closely related with protein metabolism and many steps in the urea cycle and excretion, including: protein catabolism, converting ammonia (NH_3_) to urea, transportation of ammonia through the blood stream and disposal of NH_3_. The *SIAH1* gene is related to ubiquitin-dependent protein catabolic process (GO:0006511). These pathways result in the breakdown of unusable cellular proteins into amino acids and NH_3_, allowing cells to utilize the amino acids to generate vital proteins or energy [61]. The gene *SLC24A4* has an assigned function in ion transport (GO:0006811) which involves movement of charged molecules into and out of the cell. Ammonia exists as ammonium ions (NH4^+^) at the physiological pH. The regulation of this gene is vital for efficient removal of NH4^+^ formed inside cells as a result of protein digestion. This process facilitates efficient transportation of NH4^+^ molecules into the liver where they can be converted to urea which is otherwise toxic. The *E2F7* gene has a function related to hepatocyte differentiation (GO:0070365). Hepatocytes are the functional units of the liver and this gene regulates the generation of specialized hepatocytes from unspecialized cells. The process of hepatocyte differentiation is vital for efficient conversion of NH_3_ to urea in the liver. ASS1 is known to regulate the urea cycle of animals (GO:0000050). The overall reaction involves conversion of NH_3_ into urea and the urea cycle primarily takes place in the liver. A mutation in the ASS1 gene is associated with citrullinemia, which is an autosomal recessive urea cycle disorder that causes ammonia to accumulate in the blood causing lethal reaction in newly-born Holstein Friesian calves [62]. The *GMDS* gene is involved with the GDP-mannose metabolic process (GO:0019673). One of the key purposes of the cellular metabolism is to eliminate nitrogenous waste from the body. Variations in this gene are associated with milk fatty acid traits in Canadian Holstein dairy cows [49], however, there are no previous reports on its association with milk urea production. The legumain gene has a biological function related to the renal system process which is linked to disposal of nitrogenous waste products through the kidney (GO:0003014). The suggestive genes associated with MU have similar biological functions as genes associated with the trait at genome-wide significant level. None of the genes for MU found in this study have previously been reported. Bouwman et al. [14] reported that the QTLs in chromosome 1, 6, 21 and 23 were associated with MU and MU yield in Dutch Holstein Friesian cows. Strucken et al. [11] demonstrated that the markers in chromosome 3, 13 and 27 were in association with milk urea nitrogen (MUN) and MUN content in German Holstein Friesian cows. Pegolo et al. [15] showed that markers at chromosome 4, 5 and 13 were associated with MUN in Italian Brown Swiss cows. The QTLs identified as being associated with MU in these studies are found on chromosomes associated with MU in the current study. Genes identified in the current study as being associated with MU are involved with regulating protein metabolism and N excretion. These findings suggest that MU can be genetically manipulated by controlling genes related to different stages of the protein cycle and functions of the organs associated with the excretory system.

The gene *MAP3K1*, which was identified as a candidate gene associated with ECPU, is involved with protein phosphorylation (GO:0006468). Phosphorylation is a post-translational modification that proteins undergo, is responsible for their stability and is a process that can alter the mechanical properties of milk [63]. Using GWAS, Jiang et al. [64] identified *MAP3K1* as a promising candidate gene affecting yields of milk, fat, protein, and percentages of fat and protein in Chinese Holstein cows. Genome-wide association studies also found that the *MAP3K1* gene is associated with human breast cancer [65]. Therefore, this gene probably plays an important role in keeping the bovine mammary gland function healthy [64]. The suggestive genes associated with ECPU have biological functions related to production, modification, metabolism and transportation of either milk protein or fat. However, only few of the genes have previously been reported in GWA studies. *MAP4K4* gene has been reported to be associated with milk yield, protein percentage, and mastitis susceptibility in Chinese Holstein cattle [66]. Although the association of *GGA3* gene with feed use efficiency in dairy cows has not been previously reported, its association with average daily gain and average daily feed intake has been reported in chickens [67]. The TRIM63 gene has been identified with its association to lactation persistency in Canadian Holstein cattle [57] while the *CDS1* gene is known to affect the yields of milk and fat and percentage of protein in Italian sheep [68]. Although this study reports some potential candidate genes for protein utilization efficiency in lactating dairy cows, no literature was found reporting candidate genes for ECPU in dairy cows.

Milk urea is a by-product of protein metabolism and is an indication of inefficient protein use. In the current study it was demonstrated that genes related to protein metabolism were associated with MU and that genes related to milk protein production were associated with ECPU. Efficiency of crude protein utilization is related to protein intake and to milk protein yield. The observations made in this study provide evidence that the known phenotypic relationships between MU, ECPU and CPP traits are likely to be at least partially driven by genetic differences between cows.

In our previous study using the same cows [69], highly positive genetic correlations were estimated between ECPU and yields of milk, crude protein, and fat, throughout the lactation. Those findings, together with the observations made in this study that genes associated with ECPU are also related to yields of milk, crude protein and fat, suggest that there are good candidate genes for marker-assisted selection for production efficiency in New Zealand dairy cows.

## 5. Conclusions

The present study performed GWAS using 50K SNP-chips and reported some QTLs and genes for FP, CPP, MU and ECPU. These traits showed moderate to very low trait heritabilities using either single-locus or single-step Bayesian methods. The identification of the associations of traits in the current study with the single-locus analysis was likely limited by the small sample size. However, the Bayesian method was more sensitive and effective in detecting small associations. The study reported novel QTLs and genes affecting MU and ECPU and confirmed the previously reported candidate genes and QTLs for FP and CPP. The novel candidate genes found in this study could be potentially important as commercial molecular markers for marker-assisted selection. Selection for ECPU could have a substantial impact on the economy and cow wellbeing, while selection for reduced MU might ensure environmental sustainability. Validation of the results of this study using a larger dataset containing New Zealand dairy cows is essential in order to confirm the findings before implementing them into industry breeding programs.

## Figures and Tables

**Table 1:** Cows from each farm, breed group and production season used for the genome-wise association study.

		Production Season			
Farm	Breed	Only 2016–2017	Only 2017–2018	Both Seasons	Total
Dairy 1	Friesian	17	13	49	79
	Jersey	15	18	40	73
	F × J crossbred	31	26	105	162
Dairy 4	Friesian	28	76	23	127
	Jersey	1	2	2	5
	F × J crossbred	91	32	65	188

Only 2016–2017 = number of cows milking only in season 2016–2017; only 2017–2018 = number of cows milking only in season 2017–2018; both seasons = number of cows milking in both 2016–2017 and 2017–2018 seasons.

**Table 2:** Number of observations (N), mean, standard deviation (SD), coefficient of variation (CV), minimum (Min) and maximum (Max) values of milk percentage traits, milk urea concentration and efficiency of crude protein utilization in grazing dairy cows at Massey University Dairy 1 and Dairy 4.

Trait	N	Mean	SD	CV (%)	Min	Max
FP, %	6618	5.07	0.92	18	1.77	9.77
CPP, %	6618	4.05	0.55	14	2.72	7.66
MU, mg/dL	1866	25.6	8.31	32	6.08	61.7
ECPU, %	1866	24.4	6.65	27	4.00	45.5

FP = fat percentage, CPP = crude protein percentage, MU = milk urea concentration, ECPU = efficiency of crude protein utilization.

**Table 3:** Estimates of variance components, heritabilities and repeatabilities of milk percentage traits, milk urea and efficiency of crude protein utilization using a univariate repeatability animal model in grazing dairy cows at Massey University Dairy 1 and Dairy 4.

Trait	σ^2^_a_	σ^2^_pw_	σ^2^_pa_	σ^2^_e_	σ^2^_p_	h^2^	t
FP	0.13	0.00	0.06	0.18	0.37	0.35	0.51
CPP	0.05	0.00	0.00	0.03	0.08	0.62	0.63
MU	6.77	0.68	1.73	15.29	24.47	0.28	0.38
ECPU	0.57	0.00	0.49	26.18	27.25	0.02	0.04

FP = fat percentage, CPP = crude protein percentage, MU = milk urea concentration, ECPU = efficiency of crude protein utilization, σ^2^_a_ = additive genetic variance, σ^2^_pw_ = within lactation permanent environment variance, σ^2^_pa_ = across lactation permanent environment variance, σ^2^_e_ = residual variance, σ^2^_p_ = phenotypic variance (σ^2^_a_ + σ^2^_pw_ + σ^2^_pa_ + σ^2^_e_), h^2^ = trait heritability (σ^2^_a_/σ^2^_p_), t = repeatability ([σ^2^_a_ + σ^2^_pw_ + σ^2^_pa_]/σ^2^_p_).

**Table 4:** Estimates of variance components of milk yield percentage traits, milk urea concentration and efficiency of crude protein utilization using a univariate single-step Bayesian (Bayes C, π = 0.997) linear mixed model in grazing dairy cows.

Trait	σ^2^_g_	σ^2^_m_	σ^2^_pw_	σ^2^_pa_	σ^2^_e_	σ^2^_p_	_S_^2^	h^2^	t
FP	0.07	0.13	0.00	0.04	0.18	0.42	0.31	0.48	0.57
CPP	0.02	0.03	0.00	0.00	0.03	0.08	0.38	0.63	0.63
MU	6.64	3.37	0.41	0.53	15.58	26.53	0.13	0.38	0.41
ECPU	0.17	0.02	0.00	0.58	25.16	25.93	0.00	0.01	0.03

FP = fat percentage, CPP = crude protein percentage, MU = milk urea concentration, ECPU = efficiency of crude protein utilization, σ^2^_g_ = additional polygenic variance, σ^2^_m_ = additive genetic variance explained by the markers, σ^2^_pw_ = within lactation permanent environment variance, σ^2^_pa_ = across lactation permanent environment variance, σ^2^_e_ = residual variance, σ^2^_p_ = phenotypic variance (σ^2^_g_ + σ^2^_m_ + σ^2^_pw_ + σ^2^_pa_ + σ^2^_e_), _S_^2^ = genomic heritability (σ^2^_m_/σ^2^_p_), h^2^ = trait heritability ([σ^2^_g_ + σ^2^_m_]/σ^2^_p_), t = repeatability ([σ^2^_g_ + σ^2^_m_ + σ^2^_pw_ + σ^2^_pa_]/σ^2^_p_).

**Table 5:** The single nucleotide polymorphisms (SNPs) which reached significance in single-locus association for fat percentages at genome-wide threshold (*p* < 1 × 10^−6^).

Trait	Locus	Chr	Position	*p*-Value	Effect	Effect SE	Ref	MA	MAF	Gene
FP	rs109421300	14	1,801,116	4.31 × 10^−8^	−0.06	0.01	C	T	0.43	*DGAT1*
	rs137071126	14	1,765,835	5.45 × 10^−8^	−0.06	0.01	G	C	0.45	*SLC52A2*

FP = fat percentage, Chr = chromosome, Ref = reference allele, MA = minor allele, MAF = minor allele frequency, *DGAT1* = diacylglycerol O-acyltransferase 1, *SLC52A2* = solute carrier family 52 member 2.

**Table 6:** The 1 Mb SNP windows surpass genome-wide significance level that is proportion of genetic variance (PVE) at 0.37% with their and window posterior probability of association (WPPA) for milk fat (FP) and crude protein (CPP) percentages, milk urea (MU) and efficiency of crude protein utilization (ECPU).

Trait	Window	Chr	Start–End Window (Mb)	Start SNP	End SNP	No. of SNP	PVE (%)	WPPA	Gene
FP	1506	14	1–2	1,118,964	1,971,143	22	38.12	1.00	*DGAT1*
	633	5	9.3–9.4	93,005,014	93,995,487	34	3.29	0.86	*MGST1*
	1602	15	1.2–1.3	12,020,185	12,928,912	18	0.53	0.30	-
	2418	27	3.6–3.7	36,075,350	36,959,262	16	0.53	0.18	*GPAT4*
	776	6	11.4–11.5	114,134,405	114,987,655	15	0.52	0.30	*ACOX3*
	2015	20	50–51	50,067,711	50,999,239	22	0.44	0.22	-
	2017	20	52–53	52,011,466	52,897,607	13	0.41	0.28	-
	1524	14	19–20	19,001,879	19,993,440	20	0.37	0.22	*CEBPD*
CPP	1506	14	1–2	1,118,964	1,971,143	22	9.83	1.00	*DGAT1*
	749	6	87–88	87,022,091	87,996,364	43	4.66	0.90	*CSN1S1*, *CSN2*, *CSN1S2*, *CSN3*
	299	3	2–3	2,120,827	2,903,624	15	3.20	0.68	-
	1080	9	71–72	71,021,904	71,970,552	19	2.08	0.56	*RPS12*
	2193	23	22–23	22,080,127	22,916,565	14	2.03	0.54	-
	1957	19	57–58	57,128,225	57,980,697	20	0.95	0.36	*GRIN2C*
	615	5	75–76	75,013,265	75,993,374	26	0.79	0.44	*EIF3D*
	928	8	33–34	33,060,034	33,937,052	17	0.79	0.46	-
	1945	19	45–46	45,094,650	45,989,813	18	0.50	0.20	*ADAM11*, *GOSR2*
	301	3	4–5	4,151,051	4,996,345	19	0.46	0.20	-
	700	6	38–39	38,019,605	38,939,012	49	0.45	0.30	*HERC6*
	498	4	79–80	79,008,823	79,904,993	15	0.43	0.20	-
	1499	13	79–80	79,008,708	79,991,041	20	0.41	0.22	-
	1787	17	29–30	29,008,821	29,936,157	19	0.41	0.24	-
	2045	21	8–9	8,031,396	8,955,497	19	0.41	0.18	*IGFR1*
	2347	26	17–18	17,042,328	17,986,547	21	0.41	0.24	-
	309	3	12–13	12,112,945	12,941,656	20	0.40	0.24	-
	750	6	88–89	88,049,208	88,958,861	29	0.40	0.18	-
	2263	24	39–40	39,013,392	39,987,594	24	0.40	0.22	-
	647	5	107–108	107,190,274	107,946,258	17	0.38	0.22	-
MU	2222	23	51–52	51,058,024	51,991,897	29	3.25	0.70	*GMDS*
	546	5	6–7	6,013,434	6,976,839	16	1.12	0.40	*E2F7*
	1343	12	15–16	15,017,263	15,988,893	22	1.04	0.36	*SIAH1*
	1689	16	13–14	13,015,545	13,976,725	19	0.95	0.28	-
	2505	29	30–31	30,075,012	30,986,595	17	0.86	0.30	-
	666	6	4–5	4,068,561	4,868,243	18	0.67	0.26	-
	2094	21	57–58	57,094,715	57,948,571	86	0.65	0.42	*SLC24A4*, *LGMN*
	735	6	73–74	73,015,703	73,990,002	21	0.64	0.30	-
	1320	11	100–101	100,018,300	100,942,106	18	0.64	0.20	*ASS1*
	749	6	87–88	87,022,091	87,996,364	43	0.62	0.44	-
	2314	25	27–28	27,028,950	27,987,306	23	0.6	0.30	-
	2140	22	31–32	31,020,826	31,942,134	16	0.59	0.24	-
	746	6	84–85	84,035,488	84,986,240	28	0.57	0.24	-
	2397	27	15–16	15,006,739	15,961,806	18	0.41	0.18	-
	2175	23	4–5	4,016,329	4,980,889	17	0.40	0.18	-
	364	3	67–68	67,096,213	67,987,280	14	0.39	0.22	-
	1770	17	12–13	12,057,061	12,986,781	17	0.39	0.16	-
	2481	29	6–7	6,141,144	6,976,219	13	0.39	0.14	-
ECPU	1987	20	22–23	22,006,676	22,960,402	99	0.41	0.30	*MAP3K1*

Chr = chromosome, *DGAT1* = diacylglycerol O-acyltransferase 1, *GPAT4* = glycerol-3-phosphate acyltransferase 4, *MGST1* = microsomal glutathione S-transferase 1, *ACOX3* = acyl-CoA oxidase 3 pristanoyl, and *CEBPD* = CCAAT enhancer binding protein delta, *CSN1S1* = α-S1-casein *CSN2* = β-casein, *CSN1S2* = α-S2-casein, CSN3 = kappa-casein, *RPS12* = Bos taurus ribosomal protein S12, *GRIN2C* = glutamate ionotropic receptor NMDA type subunit 2C, *EIF3D* = eukaryotic translation initiation factor 3 subunit D, *ADAM11* = ADAM metallopeptidase domain 11, *GOSR2* = golgi SNAP receptor complex member 2, *HERC6* = HECT and RLD domain containing E3 ubiquitin protein ligase family member 6, *IGF1R* = insulin-like growth factor 1 receptor, *GMDS* = GDP-mannose 4,6-dehydratase, *E2F7* = E2F transcription factor 7, *SIAH3* = solute carrier family 52 member 2, *SLC24A4* = solute carrier family 24 member 4, *LGMN* = legumain, *ASS1* = argininosuccinate synthase 1, *MAP3K1* = mitogen-activated protein kinase kinase 1.

## Supplementary Materials

The following are available online at https://www.mdpi.com/2073-4425/12/3/456/s1, Table S1: The SNPs which reached significance in single-locus association for fat percentages at suggestive threshold (*p* < 2.22 × 10^−5^), Table S2: The 1 Mb SNP windows surpass suggestive significance level that is proportion of genetic variance (PVE%) at 0.19% and their window posterior probability of association (WPPA) for percentages of milk fat (FP) and crude protein (CPP), milk urea (MU) and efficiency of crude protein utilization (ECPU).
