# Supplementary material for: Identification of Genomic Regions Associated with Concentrations of Milk Fat, Protein, Urea and Efficiency of Crude Protein Utilization in Grazing Dairy Cows
Source: Genes (Basel). 2021 Mar 23;12(3):456. doi: 10.3390/genes12030456 (PMC8004844; doi:10.3390/genes12030456)
Supplement: Supplementary file 1 [file genes-12-00456-s001.pdf]

Table S1. The SNPs which reached significance in single-locus association for fat percentages at suggestive threshold (P < 2.22 × 10-5).

Trait	Locus	Chr	Position	P-Value	Effect	Effect SE	Ref	MA	MAF	Gene	Gene function
FP	rs137787931	14	1,880,378	$4.27E^{-07}$	-0.06	0.01	T	C	0.42	*MROH1*	-
	rs134432442	14	1,736,599	$8.69E^{-07}$	0.06	0.01	C	T	0.49	*CPSF1*	mRNA polyadenylation

FP = fat percentage, Chr = chromosome, Ref = reference allele, MA = minor allele, MAF = minor allele frequency, *MROH1* = maestro heat like repeat family member 1, *CPSF1* = cleavage and polyadenylation specific factor 1.

Table S2. The 1-Mb SNP windows surpass suggestive significance level that is proportion of genetic variance (PVE) at 0.19 % and their window posterior probability of association (WPPA) for percentages of milk fat (FP) and crude protein (CPP), milk urea (MU) and efficiency of crude protein utilization (ECPU).

Trait	Window	Chr	Start-end window (Mb)	Start SNP	End SNP	No. of SNP	PVE (%)	WPPA	Gene	Gene function
FP	1849	18	15-16	15,039,844	15,954,290	21	0.31	0.22	*GPT2*	Regulation of biosynthesis
CPP	323	3	26-27	26,020,004	26,925,312	18	0.36	0.22	*TRIM45*	Protein ubiquitination
	1567	14	62-63	62,081,472	62,960,995	17	0.34	0.12	*UBR5*	Protein ubiquitination
MU	563	5	23-24	23,019,369	23,949,571	19	0.34	0.08	*UBE2N*	Protein ubiquitination
	1084	9	75-76	75,026,578	75,935,285	19	0.33	0.14	*TNFAIP3*	Protein ubiquitination
	1677	16	1-2	1,033,239	1,972,109	24	0.32	0.14	*ATP2B4,*<br>*REN*	Urinary bladder smooth muscle contraction,<br>Kidney development
	4	1	3-4	3,079,342	3,987,104	19	0.31	0.18	*UBR1*	Protein catabolic process
	399	3	102-103	102,000,000	103,000,000	20	0.25	0.1	*ATP6V0B*	Ion transport
	2125	22	16-17	16,024,708	16,983,046	16	0.23	0.12	*VHL*	Protein catabolic process
	145	1	144-145	144,000,000	145,000,000	21	0.22	0.14	*TRPM2*	Ion transport
	1440	13	20-21	20,006,828	20,982,977	17	0.22	0.14	*ARL5B*	Intracellular protein transport
	2503	29	28-29	28,078,118	28,887,293	17	0.22	0.12	*PANX3*	Cation transport
	573	5	33-34	33,078,266	33,991,103	12	0.21	0.16	*SLC38A4,*<br>*SLC38A2,*<br>*SLC38A1*	Amino acid transport<br>Ion transport<br>Amino acid transport
	1177	10	62-63	62,036,795	62,853,400	18	0.21	0.1	*SLC12A1*	Ion transport
	1775	17	17-18	17,027,443	17,947,446	13	0.2	0.1	*UCP1*	Ion transport
	1956	19	56-57	56,104,218	56,944,557	84	0.2	0.18	*NUP85,*<br>*OTOP2,*	Nephron development<br>Ion transport

									OTOP3,	Amino acid transport
									GRIN2C,	Excitatory postsynaptic potential
									SLC9A3R1	Regulation of excretion
ECPU	1230	11	10-11	10,037,282	10,985,610	15	0.3	0.08	*MAP4K4,*	Protein phosphorylation
									MRPS9,	Translation
									TGFBRAP1	Intracellular protein transport
	1956	19	56-57	56,104,218	56,944,557	84	0.3	0.16	*GGA3,*	Protein transport
									MRPS7	Ribosomal small subunit assembly
	287	2	127-128	127,000,000	128,000,000	77	0.28	0.2	*TRIM63*	Protein ubiquitination
	2210	23	39-40	39,021,955	39,980,694	80	0.26	0.22	*RNF144B*	Protein ubiquitination
	229	2	69-70	69,014,623	69,939,162	13	0.25	0.06	*INSIG2*	Lipid metabolic process
	2141	22	32-33	32,008,861	32,990,568	19	0.24	0.04	*UBA3*	Protein modification
	761	6	99-100	99,028,913	99,992,455	18	0.23	0.08	*CDS1*	Lipid metabolism
	1106	9	97-98	97,022,238	97,944,712	19	0.22	0.06	*PRKN*	Protein ubiquitination
	1207	10	92-93	92,011,190	92,935,729	17	0.22	0.06	*SEL1L*	Protein secretion
	152	1	151-152	151,000,000	152,000,000	14	0.19	0.04	*PIK3R4*	Protein phosphorylation,
	1907	19	7-8	7,004,150	7,972,717	26	0.19	0.06	*GDF6,*	Fat cell differentiation
									TRIM25	Ubiquitin-dependent protein catabolic process
	2473	28	45-46	45,096,626	45,997,628	16	0.19	0.08	*COG2*	Protein transport

Chr = chromosome, *GPT2* = glutamic-pyruvic transaminase 2, *TRIM45*= tripartite motif containing 45, *UBR5* = ubiquitin protein ligase E3 component n-recognin 5, *UBE2N* = ubiquitin conjugating enzyme E2N, *TNFAIP3* = TNF alpha induced protein 3, *ATP2B4* = ATPase plasma membrane Ca2^{+} transporting 4, *REN* = renin, *UBR1* = ubiquitin protein ligase E3 component n-recognin 1, *ATP6V0B* = ATPase H+ transporting V0 subunit b, *VHL* = von Hippel-Lindau tumor suppressor, *TRPM2* = transient receptor potential cation channel subfamily M member 2, *ARL5B* = ADP ribosylation factor like GTPase 5B, *PANX3* = pannexin 3, *SLC38A4* = solute carrier family 38 member 4, *SLC38A2* = solute carrier family 38 member 2, *SLC38A1* = solute carrier family 38 member 1, *SLC12A1*= solute carrier family 12 member 1, *UCP1* = uncoupling protein 1, *NUP85* = nucleoporin 85, *OTOP2* = otopetrin 2, *OTOP3* = otopetrin 3, *GRIN2C* = glutamate ionotropic receptor *NMDA* type subunit 2C, *SLC9A3R1* = SLC9A3 regulator 1, *MAP4K4* = mitogen-activated protein kinase kinase kinase kinase 4, *MRPS9* = mitochondrial ribosomal protein S9, *TGFBRAP1* = transforming growth factor beta receptor associated protein 1, *GGA3* = golgi-associated, gamma adaptin ear containing, ARF binding protein 3, *MRPS7* = mitochondrial ribosomal protein S7, *TRIM63* = tripartite motif containing 63, *RNF144B* = ring finger protein 144B, *INSIG2* = insulin induced gene 2, *UBA3* = ubiquitin like modifier activating enzyme 3, *CDS1* = CDP diacylglycerol synthase 1, *PRKN* = parkin RBR E3

ubiquitin protein ligase, *SEL1L* = SEL1L adaptor subunit of ERAD E3 ubiquitin ligase, *PIK3R4* = phosphoinositide-3-kinase regulatory subunit 4, *GDF6* = growth differentiation factor 6, *TRIM25* = tripartite motif containing 25, *COG2* = component of oligomeric golgi complex 2.
